# Recombinant Human Myelin-Associated Glycoprotein Promoter Drives Selective AAV-Mediated Transgene Expression in Oligodendrocytes

**DOI:** 10.3389/fnmol.2016.00013

**Published:** 2016-02-23

**Authors:** Georg von Jonquieres, Dominik Fröhlich, Claudia B. Klugmann, Xin Wen, Anne E. Harasta, Roshini Ramkumar, Ziggy H. T. Spencer, Gary D. Housley, Matthias Klugmann

**Affiliations:** Translational Neuroscience Facility and Department of Physiology, School of Medical Sciences, UNSW AustraliaSydney, NSW, Australia

**Keywords:** oligodendroglia, leukodystrophy, gene therapy, AAV, white matter disorders, myelin-associated glycoprotein

## Abstract

Leukodystrophies are hereditary central white matter disorders caused by oligodendrocyte dysfunction. Recent clinical trials for some of these devastating neurological conditions have employed an *ex vivo* gene therapy approach that showed improved endpoints because cross-correction of affected myelin-forming cells occurred following secretion of therapeutic proteins by transduced autologous grafts. However, direct gene transfer to oligodendrocytes is required for the majority of leukodystrophies with underlying mutations in genes encoding non-secreted oligodendroglial proteins. Recombinant adeno-associated viral (AAV) vectors are versatile tools for gene transfer to the central nervous system (CNS) and proof-of-concept studies in rodents have shown that the use of cellular promoters is sufficient to target AAV-mediated transgene expression to glia. The potential of this strategy has not been exploited. The major caveat of the AAV system is its limited packaging capacity of ~5 kb, providing the rationale for identifying small yet selective recombinant promoters. Here, we characterize the human myelin associated glycoprotein (*MAG*) promoter for reliable targeting of AAV-mediated transgene expression to oligodendrocytes *in vivo*. A homology screen revealed highly conserved genomic regions among mammalian species upstream of the transcription start site. Recombinant AAV expression cassettes carrying the cDNA encoding enhanced green fluorescent protein (GFP) driven by truncated versions of the recombinant *MAG* promoter (2.2, 1.5 and 0.3 kb in size) were packaged as cy5 vectors and delivered into the dorsal striatum of mice. At 3 weeks post-injection, oligodendrocytes, neurons and astrocytes expressing the reporter were quantified by immunohistochemical staining. Our results revealed that both 2.2 and 1.5 kb *MAG* promoters targeted more than 95% of transgene expression to oligodendrocytes. Even the short 0.3 kb fragment conveyed high oligodendroglial specific transgene expression (>90%) *in vivo*. Moreover, cy5-*MAG2.2-GFP* delivery to the neonate CNS resulted in selective GFP expression in oligodendrocytes for at least 8 months. Broadly, the characterization of the extremely short yet oligodendrocyte-specific human *MAG* promoter may facilitate modeling neurological diseases caused by oligodendrocyte pathology and has translational relevance for leukodystrophy gene therapy.

## Introduction

Recombinant adeno-associated virus (AAV) is the preferred vector platform for gene delivery to the central nervous system (CNS) due to its minimal potential to elicit immune response, episomal localization of the vector genome and long-term transgene expression. The major restriction of the system is the small DNA packaging limit of 4.7 kb (Dong et al., 1996).

The AAV serotype-specific tropism depends on interactions between viral capsid proteins and specific receptors at the surface of host cells and transduction is determined by intracellular processing of AAV virions (Xiao et al., 2012). Finally, transgene expression is controlled by recruitment of host cell-derived transcription factors to the recombinant promoters. Direct AAV-mediated DNA transfer to neurons has proven beneficial in conditions with a primary defect in this cell population (Weinberg et al., 2013). In addition, some leukodystrophies, central white matter disorders caused by genetic deficiencies in oligodendrocyte proteins, have been trialled successfully by *ex vivo* gene therapy acting via cross-correction of dysfunctional oligodendrocytes by uptake of graft-derived, secreted transgene products (Cartier et al., 2009; Biffi et al., 2013). With the exception of Canavan Disease and its models, neurotropic AAV vectors (Klugmann et al., 2005a; Leone et al., 2012; Ahmed et al., 2013) and *ex vivo* gene therapy approaches are blunt tools for treating leukodystrophies caused by mutations in genes encoding non-secreted proteins.

The traditional view that AAV is strictly neurotropic has been based on observations of specific neuronal transgene expression driven by viral or hybrid promoters (Fitzsimons et al., 2002). Preclinical proof-of-concept studies showing successful modification of the AAV system towards selective transgene expression in oligodendrocytes used “neurotropic” serotypes but employed the promoter of the mouse myelin basic protein (*Mbp*) gene (Chen et al., 1998; Lawlor et al., 2009; von Jonquieres et al., 2013). These findings support a promoter-swapping strategy for oligodendrocyte-targeted transgene expression with AAV gene delivery.

While the *Mbp* promoter holds promise for potential clinical applications, its murine origin, relative big size and poor specificity following neonatal vector delivery, are potential caveats (von Jonquieres et al., 2013).

The aim of the present study was to characterize a human oligodendrocyte-specific promoter suitable for reliable AAV-mediated transgene expression *in vivo*. In the CNS, myelin-associated glycoprotein (MAG) is a pre-myelinating marker responsible for oligodendroglial recognition of axons and myelin maintenance (Martini and Schachner, 1997). These features provided the rationale for investigating the potential of the *MAG* gene promoter for directed AAV-mediated transgene expression in oligodendrocytes. Employing a bioinformatics approach, we identified a 2.2 kb region upstream of the putative transcription start site of the human *MAG* promoter that comprised two areas that were highly conserved across mammalian species. We then isolated the genomic DNA fragments and generated AAV plasmids expressing the enhanced green fluorescent protein (GFP) reporter under the control of either the 2.2 kb *MAG* promoter, or truncated 1.5 and 0.3 kb fragments containing both or just one conserved area, respectively. All three *MAG* promoter constructs drove GFP expression in oligodendrocytes *in vitro* as well as *in vivo* following intrastriatal infusion of the corresponding AAV vectors to adult mice. Neonatal delivery of the *MAG2.2-GFP* vector resulted in highly specific oligodendroglial expression persisting for at least 8 months. Our data suggest that the novel recombinant *MAG* promoter will be instrumental for preclinical gene function studies and clinical gene therapy alike, that require long-term and specific AAV-mediated transgene expression in oligodendrocytes.

## Materials and Methods

### Animals

C57BL/6J mice were group-housed (2–4 cage mates) in a temperature-controlled room (21–22°C; 49–55% humidity) with 12 h-light-dark-cycle (lights on 7:00–19:00), where food and water were available *ad libitum*. Experiments were approved by the UNSW Animal Care and Ethics Committee (UNSW ACEC 11/130A and 14/154B).

### Bioinformatics

We identified the human *MAG* gene locus using the UCSC genome browser^1^. Based on the March 2006 alignment, the genomic sequence from Chromosome 19q13.1: 40469878–40496547 (Chr. 19: 35289949–35292134 in the current GRCh38. p2 assembly) including exons, introns and a 5 kb upstream putative promoter region of the *MAG* locus was assessed for genomic conservation using the Vista browser^2^ (Thoms et al., 2011). The putative *MAG* promoter and in particular regions of >50% interspecies conservation were screened for transcription factor binding sites known to be relevant to the oligodendroglial linage using JASPAR^3^, Wilmer Bioinformatics^4^ or the Patch1.0 Software^5^.

### Plasmid Constructs

AAV-GFP plasmids in which reporter gene expression was controlled by the 1.1 kb cytomegalovirus (CMV) enhancer/chicken β-actin hybrid (CAG) promoter (pAAV-CAG-*GFP*), the human glial fibrillary acidic protein promoter (pAAV-*GFAP-GFP*), or the mouse myelin basic protein promoter (pAAV-*Mbp-GFP*), were generated as described previously (von Jonquieres et al., 2013).

The regions 2.2 and 1.5 kb upstream of the *MAG* transcriptional start site were PCR amplified from a genomic DNA template isolated from the human oligodendroglial cell line MO3.13 using specific primers (MAG_2.2 kb_fwd: cctcagaaggaaccaacactgccag; MAG_1.5 kb_Fwd: cgactccagctccaactagg; MAGrev: gcccccacttgccagcccctcccct). The PCR products were subcloned into the XhoI and AgeI sites of pAAV-*Mbp-GFP* to replace the *Mbp* promoter, generating pAAV-*MAG2.2-GFP* and pAAV-*MAG1.5-GFP*. For truncation of the *MAG* promoter down to 0.3 kb, pAAV-*MAG1.5-GFP* was subjected to an Acc65I/Bsu36I restriction digest. Separation of a 1.2 kb fragment corresponding to the 0.3–1.5 kb distal promoter region was confirmed by agarose gel electrophoresis. The remaining 5.4 kb fragment containing the AAV-plasmid backbone, the proximal 0.3 kb *MAG* promoter and the GFP cDNA was gel-extracted, blunted by Klenow fill-in and re-ligated to obtain pAAV-*MAG0.3-GFP*. The integrity of the recombinant clones was confirmed by analytical digests and DNA sequencing.

### AAV Vector Production

AAV vector packaging was performed as described previously (Harasta et al., 2015). Briefly, human embryonic kidney 293 (HEK 293) cells were triple-transfected with the AAV-GFP plasmid, the serotype-specific AAV helper plasmid and the adenovirus helper plasmid (pFΔ6) by standard calcium phosphate transfection. Chimeric AAV1/2 vectors carrying VP1, VP2 and VP3 capsid proteins from AAV1 and AAV2 at roughly equal ratios, were produced as described following quadruple plasmid transfection (Klugmann et al., 2005b; McClure et al., 2011). All vectors were harvested 60 h after transfection, purified using iodixanol (OptiPrep^TM^, Sigma-Aldrich) gradient ultracentrifugation and concentrated 3× by refilling with phosphate-buffered saline containing 1 mM MgCl_2_ and 2.5 mM KCl using Microsep^TM^ Advanced Centrifugal Device 100 K MWCO concentrators (Pall, Surry Hills, NSW, Australia). Genomic titres were determined with primers designed to WPRE (During et al., 2003).

### AAV Vector Delivery *In Vivo*

Intraparenchymal delivery of AAV vectors into the striatum of neonatal or adult mice was performed as described (von Jonquieres et al., 2013). Briefly, 1 μl of AAV-GFP vector, adjusted to 2 × 10^12^ vector genomes (vg)/ml, was injected into the dorsal striatum. Vector delivery was performed using a microprocessor-controlled mini-pump (World Precision Instruments (WPI), Sarasota, FA, USA) with a 34G bevelled needle (WPI).

Adult mice (2–4 months; both sexes) were anesthetized with isoflurane (4% induction, then 1% maintenance with O_2_). Animals were placed into a stereotaxic frame (Kopf instruments, Tujunga, CA, USA). One microlitre of AAV-GFP vector was injected into the striatum (+1.1 mm AP, −1.7 mm ML, −3.5 mm DV from bregma). Vector delivery was performed at a rate of 150 nl/min and the needle was left in place for 5 min prior to slowly retracting it from the brain.

For neonatal vector delivery (P0), pups (8–24 h after birth) were cryo-anesthetized and AAV administered as described (Pilpel et al., 2009). Briefly, pups were immobilized by wrapping in a paper towel covered with wet-ice for 3–5 min and then positioned in a custom-made styrofoam mold for vector delivery (100 nl/s) into the striatum (+2.0 mm AP, −1.5 mm ML, −2.0 mm DV from lambda) using a hand-held needle (WPI). The needle was left in place for additional 10 s at the end of the injection to prevent backflow of virus containing solution. The pups were then re-warmed on a heating matt and rolled in the bedding of their pre-warmed home cage before being returned to the dam.

### Cell Culture

HEK 293 cells were cultured in Dulbecco’s Modified Eagle Medium with 10% fetal calf serum and 1 mM sodium pyruvate and transfected using the calcium phosphate precipitation method. The mouse oligodendroglial cell line Oli-neu was cultured in Sato with 1% horse serum and transfected by electroporation as described (Krämer et al., 1997; Frühbeis et al., 2013). Differentiation was induced by daily application of 1 mM dibutyryl cyclic adenosine monophosphate (dbcAMP). Cells were seeded at a density of 5 × 10^4^ per 11 mm glass coverslip and then kept for additional 4 days before fixation or lysis.

### Immunoblotting

Detection of the GFP antigen in HEK 293 and Oli-neu protein lysates following sodium dodecyl sulfate polyacrylamide gel electrophoresis and electroblotting was performed as described (Harasta et al., 2015). Forty eight hours (HEK 293) and 96 h (Oli-neu) post transfection, protein lysates were extracted and 10 μg of total protein was size separated and immobilized on a polyvinyl fluoride membrane. Equal loading was confirmed by staining the membrane with Ponceau S. After washing and blocking using 5% milk powder, the membranes were incubated with a rabbit anti-GFP antibody, produced in-house (von Jonquieres et al., 2013). This was followed by application of an anti-rabbit horseradish peroxidase-conjugated secondary antibody (Dianova, Hamburg, Germany), detection of the immunoreactivity by the enhanced chemiluminescence system (BioRad, Gladesville, NSW, Australia) and signal capture (GelDoc, BioRad, Gladesville, NSW, Australia).

### Immunohistochemistry

Mice were fixed by transcardial perfusion with 10% buffered neutral formalin (Sigma) and 40 μm coronal cryosections spanning the subcortical striatal nuclei (including globus pallidus and caudate putamen) were collected after cryoprotection in 30% sucrose. Tissue treatment by antigen retrieval followed by immunodetection of antigens has been described elsewhere (von Jonquieres et al., 2014). Sections were treated with a combination of primary antibodies including rabbit-anti GFP or mouse anti-GFP (Roche, Switzerland) with either mouse anti-NeuN (Millipore, MA, USA), mouse anti-glial fibrillary acidic protein (GFAP; Sigma-Aldrich, MO, USA), or rabbit anti-aspartoacylase (ASPA; Mersmann et al., 2011). ASPA is a marker of mature oligodendrocytes. In this population, ASPA positive cells have been reported to overlap 100 and 93% with the widely used oligodendrocyte soma markers glutathione S-transferase π isoform and CC1, respectively (Kawai et al., 2009). Following incubation with the appropriate Alexa-conjugated secondary antibodies (Invitrogen, Carlsbad, CA, USA), sections were mounted on slides and coverslipped with Mowiol (Calbiochem, Darmstadt, Germany). Fluorescence was visualized using a Zeiss Z1 AxioExaminer NLO710 confocal microscope (Carl Zeiss MicroImaging, Germany).

### Quantification of Cell-Type Specific Transgene Expression

Specificity of AAV-mediated transgene expression was assessed following our previous work (von Jonquieres et al., 2013). Neonates (P0), or adult mice (*n* = 3) injected with AAV were humanely killed by transcardial perfusion 3 weeks later. Some neonatally injected mice were examined at 8 months for a long-term study. The identity of GFP-expressing cells in the striatum was examined by double-immunofluorescence with antibodies against GFP and ASPA (oligodendrocytes), NeuN (neurons), or GFAP (astrocytes) in confocal images at 20× and 40× magnification. The percentage of GFP-expressing cells per cell-type was determined by counting at least 50 cells from each of three non-adjacent sections for a total of at least 150 GFP^+^ cells using the “cell counter” plugin for ImageJ version 1.45 k (NIH). GFAP is a marker, generally regarded as pan-astrocytic. However, recent evidence has shown that GFAP is heterogeneously expressed in the astroglial compartment with a bias towards white matter and reactive astrocytes (Cahoy et al., 2008). Therefore, transduced astrocytes were identified based on the presence of GFAP and morphological criteria. The latter was possible as the soluble GFP reporter filled the processes of the host cells.

### Statistics

All graphs and statistical analyses were done with GraphPad Prism 6 Software (La Jolla, CA, USA). Quantitative measures were analyzed by one-way or two-way analysis of variance (ANOVA) as appropriate, followed by Tukey’s *post hoc* test. Values are presented as the mean ± SEM.

## Results

### *Mbp* Promoter-Driven Transgene Expression in Oligodendrocytes Following Intracranial Injection of AAV1/2, rh39, rh20 and cy5

**Figure 1:**
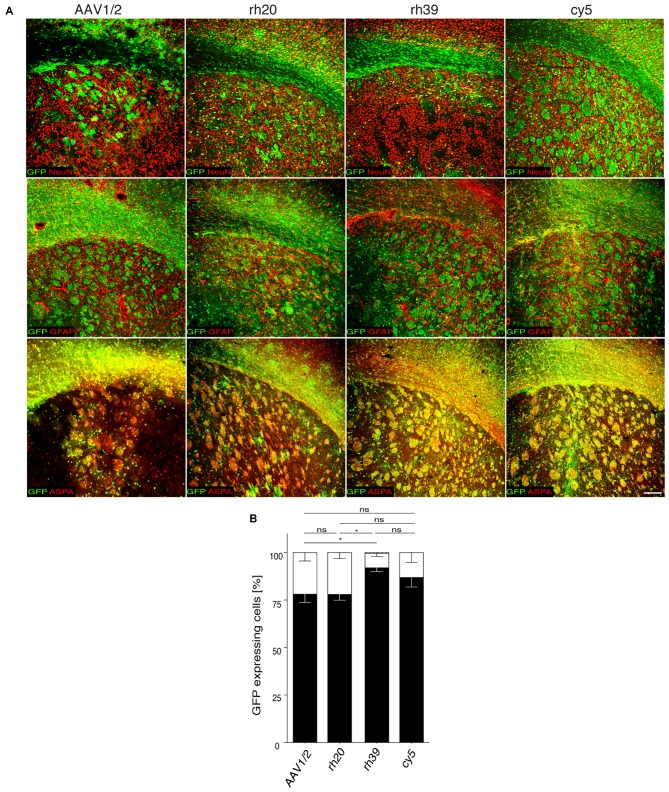
***Mbp* promoter selectivity targets transgene expression to oligodendrocytes in the adult brain. (A)** The indicated adeno-associated viral (AAV) vectors (2 × 10^9^ vg) were injected into the striatum of adult mice (*n* = 3). Representative images showing immunohistochemistry for green fluorescent protein (GFP; green) and cell type specific markers (red) including ASPA (oligodendrocytes), NeuN (neurons) and GFAP (astrocytes). **(B)** Quantification of relative GFP reporter expressing in ASPA^+^ oligodendrocytes, NeuN^+^ neurons and GFAP^+^ astrocytes. Two-way analysis of variance (ANOVA) with Tukey’s *post hoc* test revealed differences in oligodendroglial GFP expression between AAV1/2 (78.2 ± 4.4%) and rh39 (91.0 ± 1.5%; *p* < 0.05) as well as rh20 (78.0 ± 1.7%) and rh39 (*p* < 0.05). There was no significant difference between rh39 and cy5 (87.0 ± 5.1%); one-way ANOVA with Tukey’s *post hoc* test. *p < 0.05. Bar: 200 μm.

We reported previously on the efficacy of chimeric AAV1/2 for the transfer of an *Mbp* promoter driven GFP expression cassette to the developing, or adult mouse brain (von Jonquieres et al., 2013). To expand this gene delivery system towards a potential clinical setting, we screened the transduction specificity of non-chimeric AAV vector variants rh20, rh39 and cy5, derived from non-human primates (Gao et al., 2004). These vectors, expressing GFP under the control of the *Mbp* promoter, were injected into the striatum of adult mice. Chimeric AAV1/2-*Mbp-GFP* served as a control (Figure 1). Three weeks later, we determined the relative numbers of GFP^+^ cells among oligodendrocytes, neurons or astrocytes by immunohistochemistry. We found that AAV1/2 and rh20 resulted in highly preferential oligodendroglial GFP expression, exceeded by rh39 and cy5. For all other experiments we focused on cy5 as it is a variant of AAV7 that, based on its extremely weak immunogenicity, has been proposed to have a strong potential to be developed as a clinical gene therapy vector (Gao et al., 2002).

### Characterization of the Human *MAG* Promoter *In Silico*

**Figure 2:**
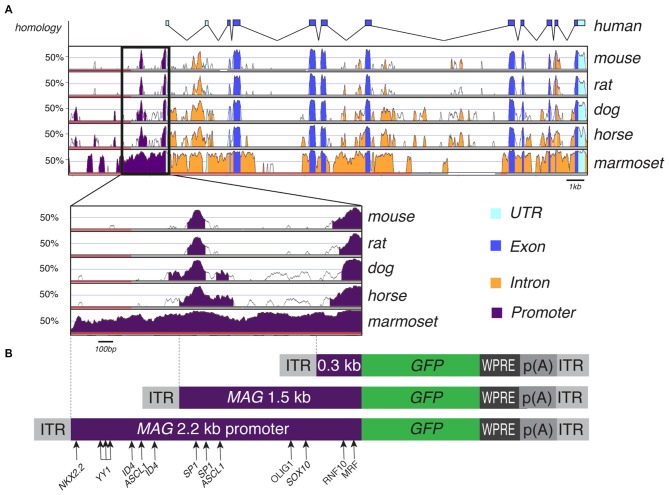
**Myelin Associated Glycoprotein (*MAG*) promoter inter-species alignment and AAV expression cassettes. (A)** VISTA plot of human sequence across the *MAG* locus and its genomic conservation in mouse, rat, dog, horse and marmoset. A close-up of the 2.2 kb upstream promoter region shows two regions of high conservation, suggestive of functional importance, from −118 to −1 bp and from −1315 to −1176 bp upstream the putative transcription start site. **(B)** AAV expression cassettes for GFP reporter expression controlled by the 0.3 kb, 1.5 and 2.2 kb human *MAG* promoter. Transcription factor binding sites with relevance to oligodendroglial gene expression are indicated.

Our *in silico* search of AAV-compatible promoter regions of oligodendrocyte-specific genes identified a human *MAG* promoter region entailing the sequence from position −2184 to the transcription start site. We located a number of known transcription factor binding sites of the oligodendrocyte lineage (Figure 2). The distal segment of this fragment contained binding sites for both positive and negative transcriptional regulators such as Yin Yang 1 (YY1) and Inhibitor of DNA Binding 4 (Id4; Marin-Husstege et al., 2006; He et al., 2007; Zolova and Wight, 2011). We therefore decided to also investigate the proximal 1.5 kb of the long fragment since this fragment was devoid of these motifs but contained binding sites for myelin cell lineage factors Oligodendrocyte Transcription Factor 1 (Olig1) and SRY-Box 10 (Sox10) and an evolutionary highly conserved sequence (Wang et al., 2014). Finally, we also selected the proximal 300 bp fragment for further characterization as it contains evolutionary conserved binding sites for myelin gene regulatory factor (MRF) and Ring Finger Protein 10 (RNF10), critical activators of myelination in oligodendrocytes or Schwann cells, respectively (Hoshikawa et al., 2008; Emery et al., 2009; Bujalka et al., 2013). Another conserved DNA stretch was located between −1315 to −1176. A comprehensive list of transcription factor binding motifs in the 2.2 kb upstream sequence of the *MAG* transcription start site is summarized in Table 1.

**Table 1:** **Position of putative transcription factor binding sites in the recombinant human *MAG* promoter**.

Transcription factor	Binding motif	Site 1	Site 2	Site 3
*MRF	ctggcac	−40 to −33	–	–
*RNF10	acaagggcccctttgtgccc	−108 to −92	–	–
*SOX10	acaatg	−293 to −287	–	–
OLIG1	tcagatg	−388 to −381	–	–
NKX2.2	acttga	−2158 to −2152	–	–
SP1	ccccctcccca	−91 to −79	−1114 to −1103	−1219 to −1208
YY1	gccatg	−1894 to −1888	−1949 to −1943	−2000 to −1994
ASCL1	cagctg	−1014 to −1008	−1715 to −1709	–
FOXD3	ttttgtttgttt	−2111 to −2099	–	–
ID4	cacctg	−1656 to −1650	−1737 to −1731	–
Zfp191	ggagggg	−735 to −728	–	–

Notes: *Transcription factor binding sites that have been experimentally validated in the rodent *MAG* promoter. See text for references.

### Recombinant *MAG* Promoter Shows Oligodendroglial Selectivity *In Vitro*

**Figure 3:**
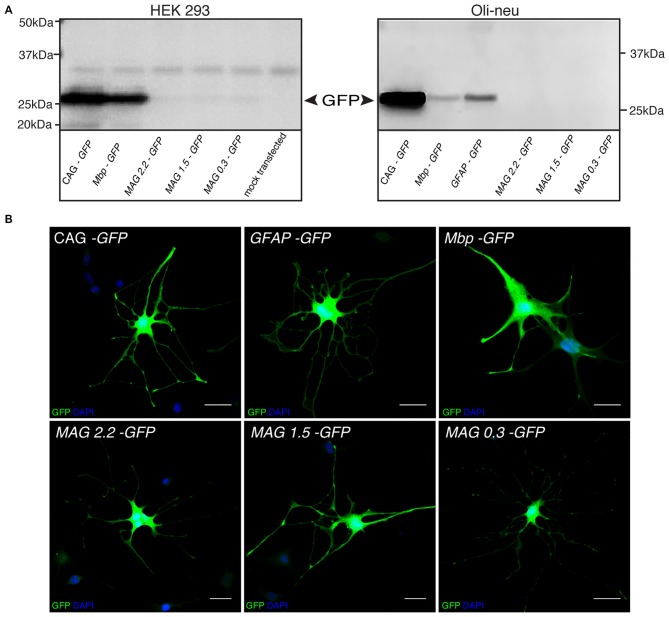
***MAG* promoter selectivity *in vitro*. (A)** GFP Immunoblots of HEK 293 and Oli-neu cells transiently transfected with the indicated plasmids. **(B)** Immunofluorescence detection of GFP expression following dbcAMP-induced differentiation of Oli-neu cells transfected with the indicated plasmids. Activity of the GFAP promoter was restricted to cells with astroglial morphology while the other promoters drove transgene expression in cells exhibiting oligodendrocyte morphology. Representative results of three independent experiments are shown. Bars: 30 μm.

We isolated three overlapping *MAG* promoter fragments stretching from position −2.2, −1.5, or −0.3 kb to the transcription start site. These fragments were inserted in an AAV2 expression cassette containing the cDNA encoding GFP (Figure 2). The recombinant AAV plasmids were then used for transient transfection of HEK 293 cells and subsequent immunocytochemistry (not shown) or immunoblot detection of the GFP reporter (Figure 3A). Low level GFP expression was detectable following transfection with the *MAG* promoter-driven constructs. In contrast, the control plasmids in which reporter gene transcription was driven by the CAG or *Mbp* promoter produced robust GFP expression. The difference between *Mbp* and *MAG* promoter activity in HEK 293 cells suggested better selectivity of the latter.

In order to substantiate this notion by positive data, we electroporated Oli-neu cells, an *in vitro* model of oligodendrocytes, with the GFP reporters controlled by either one of the three *MAG* promoter fragments, the *GFAP*, *Mbp*, or CAG promoter. The cultures were then differentiated to adopt a mature oligodendrocyte-like phenotype, and finally assessed by immunoblot or GFP-immunocytochemistry. Unexpectedly, GFP-expression was below the detection limits of the immunoblot following transfection with any of the three *MAG* promoter-GFP constructs (Figure 3A). As the transfection efficiency was generally >70% judged by CAG-GFP transfectants (not shown) this result suggested that our recombinant *MAG* promoters were hardly active in the Oli-neu model. However, at the single cell level *MAG*-GFP transfection resulted in GFP-immunoreactivity in Oli-neu with oligodendrocyte morphology (Figure 3B).

### Specificity of *MAG* Promoter Driven cy5 Vectors in the CNS of Adult Mice

**Figure 4:**
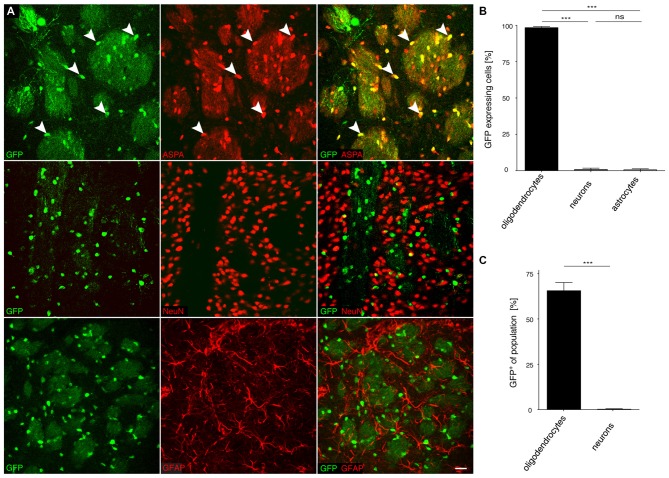
**The recombinant *MAG* 2.2 promoter drives oligodendrocyte-specific transgene expression in the central nervous system (CNS). (A)** cy5-*MAG2.2-GFP* (2 × 10^9^ vg) was injected into the dorsal striatum of adult mice 3 weeks prior to analysis (*n* = 3). Immunohistochemical staining was performed for GFP (green) and cell type specific markers (red) including ASPA (oligodendrocytes), NeuN (neurons) and GFAP (astrocytes). Arrowheads indicate representative cells showing colocalization. **(B)** Quantification of the results in **(A)** showed that GFP reporter gene expression was almost completely restricted to oligodendrocytes (98.4 ± 0.8%). Expression in neurons (0.9 ± 0.9%) and astrocytes (0.7 ± 0.67%) was negligible. **(C)** Relative quantification of the percentage of GFP expressing cells in each population revealed that 65.1 ± 4.0% of all oligodendrocytes expressed the GFP transgene while the ratio of GFP expressing neurons was extremely low (0.3 ± 0.27%); One-way ANOVA with Tukey’s *post hoc* test. ***p < 0.001. Bar: 20 μm.

**Figure 5:**
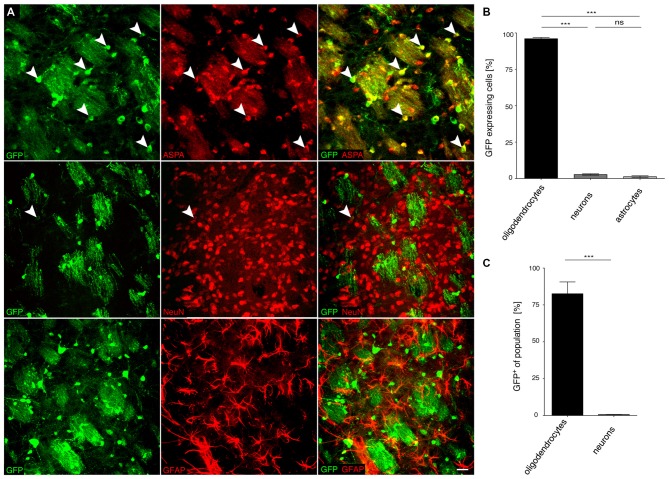
**The *MAG* 1.5 promoter restricts GFP transgene expression to oligodendrocytes. (A)** cy5-*MAG1.5-GFP* (2 × 10^9^ vg) was injected into the dorsal striatum of adult mice 3 weeks prior to analysis (*n* = 3). Immunohistochemical staining was performed for GFP (green) and cell type specific markers (red) including ASPA (oligodendrocytes), NeuN (neurons) and GFAP (astrocytes). Arrowheads indicate representative cells showing colocalization. **(B)** Quantification of the results in **(A)** showed that GFP reporter gene expression was almost completely restricted to oligodendrocytes (96.0 ± 0.9%). Expression in neurons (2.8 ± 0.5%) and astrocytes (1.3 ± 0.6%) was significantly less. **(C)** Relative quantification of the percentage of GFP expressing cells in each population revealed that 82.6 ± 7.7% of all oligodendrocytes expressed the GFP transgene while the ratio of GFP expressing neurons was extremely low (0.8 ± 0.1%); One-way ANOVA with Tukey’s *post hoc* test. ***p < 0.001. Bar: 20 μm.

**Figure 6:**
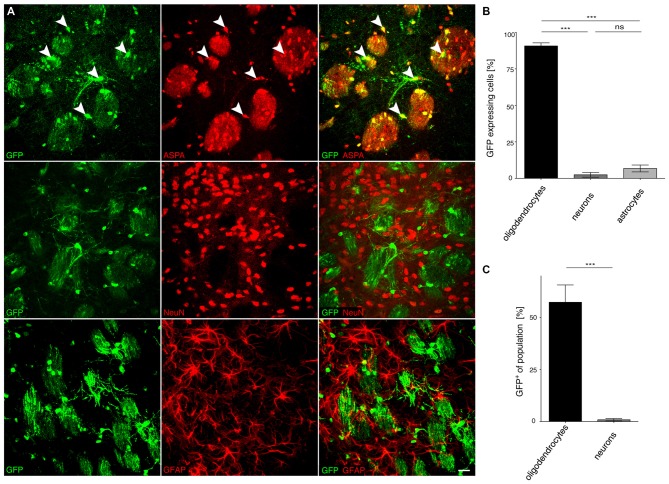
**The truncated *MAG0.3* promoter is sufficient to restrict AAV-mediated transgene expression to oligodendrocytes. (A)** cy5-*MAG0.3-GFP* (2 × 10^9^ vg) was injected into the dorsal striatum of adult mice 3 weeks prior to analysis (*n* = 3). Immunohistochemical staining was performed for GFP (green) and cell type specific markers (red) including ASPA (oligodendrocytes), NeuN (neurons) and GFAP (astrocytes). Arrowheads indicate representative cells showing colocalization. **(B)** Quantification of the results in **(A)** showed that GFP reporter gene expression was almost completely restricted to oligodendrocytes (90.7 ± 2.1%). Expression in neurons (2.5 ± 1.7%) and astrocytes (6.8 ± 2.4%) was significantly less. **(C)** Relative quantification of the percentage of GFP expressing cells in each population revealed that 57.3 ± 8.4% of all oligodendrocytes expressed the GFP transgene while the ratio of GFP expressing neurons was extremely low (0.9 ± 0.6%); One-way ANOVA with Tukey’s *post hoc* test. ***p < 0.001. Bar: 20 μm.

We then investigated the potential of the *MAG* promoter for AAV-mediated transgene expression *in vivo*. All three AAV-*MAG*-*GFP* constructs were packaged into cy5 vectors and delivered to the dorsal striatum of adult mice. At 3 weeks following vector injection, when AAV-mediated transgene expression has peaked to stable levels (Klugmann et al., 2005a), animals were killed and the brains assessed by immunohistochemistry to detect GFP in immuno-identified neurons, oligodendrocytes, and astrocytes. Quantitative analyses of cy5-*MAG2.2-GFP* specificity judged by relative numbers of GFP^+^ cells revealed a definitive oligodendroglial selectivity of the long *MAG* promoter fragment *in vivo* (Figures 4A,B). Neurons and astrocytes were almost entirely excluded from GFP-expression. Most oligodendrocytes in the target area, identified by ASPA immunoreactivity, expressed the GFP reporter (Figure 4C). Only a negligible number of neurons were GFP-positive. Similar results were obtained using cy5-*MAG1.5-GFP* (Figure 5) and cy5-*MAG0.3-GFP* (Figure 6). The use of the GFAP antibody precluded this sort of analysis for astroglia as it labels heterogeneous astrocyte populations (Cahoy et al., 2008).

### Selectivity of cy5-*MAG2.2-GFP* in the CNS of Neonatal Mice

**Figure 7:**
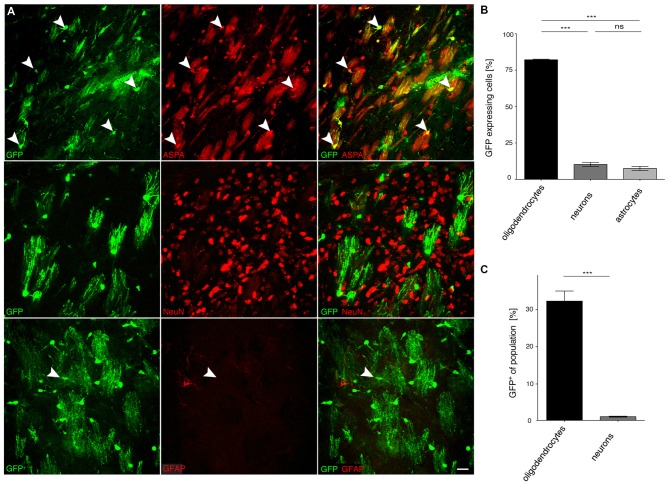
**Long-term expression in oligodendrocytes following neonatal cy5-*MAG2.2-GFP* delivery. (A)** cy5-*MAG2.2-GFP* (2 × 10^9^ vg) was injected into the dorsal striatum of neonatal mice (*n* = 4). At 8 months, immunohistochemical staining was performed for GFP (green) and cell type specific markers (red) including ASPA (oligodendrocytes), NeuN (neurons) and GFAP (astrocytes). Arrowheads indicate representative cells showing colocalization. **(B)** Quantification of the results in **(A)** showed that GFP reporter gene expression was enriched in the oligodendroglial compartment (82.2 ± 0.4%). Expression in neurons (10.3 ± 1.4%) and astrocytes (7.6 ± 1.4%) was significantly less. **(C)** Relative quantification of the percentage of GFP expressing cells in each population revealed that 32.8 ± 2.8% of all oligodendrocytes expressed the GFP transgene while the ratio of GFP expressing neurons was extremely low (1.0 ± 0.1%). ***p < 0.001. Bar: 20 μm.

Somatic transgenesis in the mouse, achieved by neonatal vector administration and transduction of target oligodendrocytes, holds potential for disease modeling and gene function studies. In the rodent forebrain, MAG expression starts at P5 (Mingorance et al., 2005) and AAV-mediated gene activation in the CNS takes several days as it requires uncoating, nuclear translocation and second-strand synthesis of the ssAAV genome. Moreover, we have previously reported a largely superior vector spread following neonatal compared with adult AAV delivery to the CNS (von Jonquieres et al., 2013). We therefore investigated the long-term effects of the *MAG* promoter in brain sections obtained 8 months after intracranial AAV-injection to neonates (Figure 7A). We selected cy5-*MAG2.2-GFP* for this experiment based on the strict oligodendroglial selectivity evident from the adult 3 week expression pattern. GFP in the striatum of animals injected neonatally was predominantly found in oligodendrocytes, but also in some neurons and astrocytes (Figure 7B). The number of GFP^+^ cells within the oligodendroglial compartments was limited (Figure 7C). A similar trend was observed in neonatally infused animals that were analyzed after 3 weeks (not shown).

**Figure 8:**
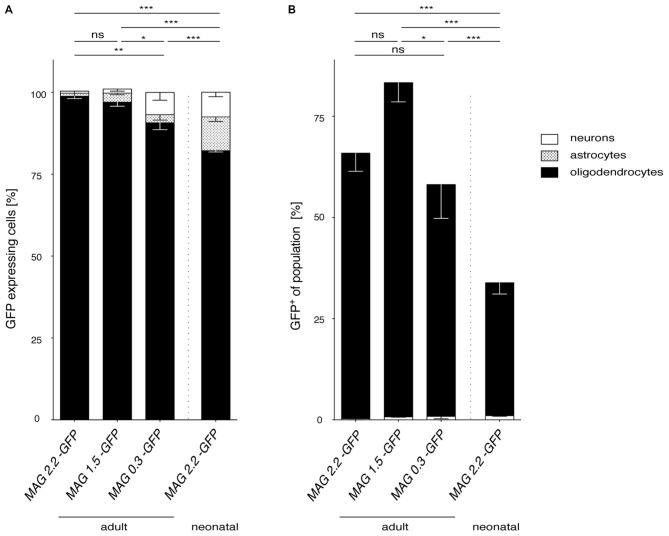
**Comparison of *MAG* promoter-controlled selectivity of AAV-mediated transgene expression. (A)** The proportion of oligodendrocytes, neurons and astrocytes was calculated for each *MAG* promoter variant in adult AAV injected mice and for cy5-*Mag2.2-GFP* delivered to neonates. Statistically significant differences in AAV-mediated oligodendrocyte-specific GFP reporter gene expression were detected between the *MAG2.2* and the *MAG0.3* promoter following AAV delivery to adult mice (*P* < 0.05). In all cases AAV mediated delivery of any of the three *MAG*-GFP expression constructs resulted in slight but significantly better restriction of transgene expression to oligodendrocytes than neonatal delivery of *MAG*2.2-*GFP* (*p* < 0.01)*.*** (B)** Summery of the percentage of GFP^+^ cells relative to the population of oligodendrocytes and neurons in the target area. Irrespective of the time point of AAV-delivery the *MAG* promoter robustly targeted the oligodendrocyte population. Adult cy5 mediated delivery of any of the three *MAG*-GFP expression constructs targeted the oligodendrocyte population significantly more efficiently than cy5-*MAG2.2-GFP* following neonatal delivery. Two-way ANOVA with Tukey’s *post hoc* test. *p < 0.05; **p < 0.01; ***p < 0.001.

Broadly, the performance of the three different MAG promoter fragments following AAV vector delivery to the CNS, summarized in Figure 8, was excellent. In the adult mouse, we observed a remarkably moderate trade-off between *MAG* promoter length and oligodendroglial selectivity. Even the short 0.3 kb fragment greatly limited transgene expression to oligodendrocytes (Figure 8A). Moreover, the 0.3 kb fragment also showed activity in the majority of oligodendrocytes in the target area comparable to the 2.2 kb fragment (Figure 8B).

## Discussion

Conventional transgenic mouse lines utilizing myelin gene-specific promoters have informed on selective cis-acting elements that were shown to restrict the expression of transgenes to myelinating glia. There is virtually no size limitation for myelin gene promoters used in transgenic mouse lines. In contrast, the somatic gene delivery system represented by AAV depends on efficient incorporation of an expression cassette of less than 4.7 kb into the confined space of the viral capsid (Warrington and Herzog, 2006).

Only a few small cellular promoters have been described to reliably drive transgene expression in oligodendrocytes in genetically modified animals. Therefore, the number of candidates to be adopted for use in viral gene transfer has been limited. Recently, the 2,3-cyclic nucleotide 3-phosphodiesterase promoter, first described in transgenic mice (Gravel et al., 1998), has been reported to convey oligodendroglial-specific GFP reporter gene expression following lentiviral delivery to the neonatal mouse brain (Kagiava et al., 2014). Although this 4 kb promoter fragment is useful in the lentiviral setting harboring vector genomes up to 8.5 kb, it does not match the AAV size criteria.

To date, the only cellular promoter used for AAV-mediated transgene expression in oligodendrocytes is the murine 1.9 kb *Mbp* cis-regulating element and a truncated version spanning the proximal 1.3 kb upstream region including parts of *Mbp* exon 1 (Chen et al., 1998; Lawlor et al., 2009; von Jonquieres et al., 2013). The *Mbp* promoters have been used independently to achieve reporter gene expression in the adult rodent CNS using AAV2, AAV8, rh39, rh20 and cy5 (Chen et al., 1998; Lawlor et al., 2009). Like the 2,3-cyclic nucleotide 3-phosphodiesterase promoter both *Mbp* promoters were selected as they had been shown to drive transgenes in the oligodendrocyte lineage in transgenic mouse lines generated by pronucleus injections (Gow et al., 1992; Orian et al., 1994).

To date there is no oligodendrocyte-specific alternative to the 1.3 and 1.9 kb *Mbp* promoter variants appropriate for AAV viral gene delivery. The goal of this investigation was to delineate a novel recombinant promoter suited for clinical gene therapy of white matter disorders. To that end, we have selected a 2.2 kb region upstream of the transcription start site in the human *MAG* gene and two truncated variants, that harbor an abundance of binding motifs for transcription factors known to regulate gene activity and myelination in oligodendrocytes or Schwann cells (Emery, 2010).

All three recombinant *MAG* promoters showed very little activity in HEK 293 cells despite good transfection efficacy confirmed to be greater than 80% in CAG-*GFP* samples. Compared with the CAG promoter, the 1.9 kb *Mbp* promoter showed weaker yet robust GFP expression in HEK 293 cells. We have, however, identified Oli-neu cells as a suitable model for qualitative observations on glial promoter selectivity given that differentiated cultures contain cells with either oligodendrocyte or astroglial morphology that were permissive for *MAG* or *GFAP* promoter driven GFP expression, respectively.

Our previous data using the *Mbp* promoter were obtained using a chimeric AAV vector system that inherently lacks translational relevance due to the heterogeneity of viral particles consisting of a wide range of ratios between AAV1 and AAV2 capsid proteins (von Jonquieres et al., 2013). Therefore, the current study employed the AAV7-derived variant cy5 as a candidate for clinical gene therapy applications since neutralizing antibodies to AAV7 are rare in human serum (Gao et al., 2002). While a recent pioneering study has confirmed very good vector spread following cy5-CAG-*GFP* delivery to the adult rodent brain the effects of cellular promoters were not examined (Lawlor et al., 2009).

We have shown previously that the vector spread depends on the developmental stage of the target tissue at the time of infusion but not on the cellular promoter used in the expression cassette (von Jonquieres et al., 2013). Thus, our current investigations focused on promoter selectivity rather than vector spread. We have previously reported that the *Mbp* promoter exhibits mostly astroglial activity following injection at P0. As this developmental stage in the mouse CNS corresponds to the second prenatal trimester in humans (Clancy et al., 2001), it is of limited clinical relevance. However, somatic transgenesis in the mouse, achieved by vector delivery at P0, is instrumental for disease modeling (Dayton et al., 2012). Therefore, we selected cy5-*MAG2.2-GFP* for intracranial injection at P0 and noticed GFP expression mostly in oligodendrocytes for at least 8 months.

Although a comparison between the transduction profiles of the vectors carrying the *MAG* promoter (this study) and the *Mbp* promoter (von Jonquieres et al., 2013) is not feasible, it appears that in neonates the *MAG* promoter might be better suited for transgene expression targeted to oligodendrocytes (90%) than the *Mbp* promoter (25%). We hypothesized that the potential gene delivery to oligodendrocyte precursor cells (OPCs) available at P0, a time point preceding MAG expression, would prime the transduced cells for MAG promoter-driven transgene expression at later stages as the recombinant AAV-genome stably remains in the host cell nucleus. However, we observed only a moderate total number of GFP-expressing oligodendrocytes following neonatal injections which might reflect a poor initial transduction efficiency of OPCs and limited available AAV particles when oligodendrocytes are born.

To our best knowledge, this is the first report on a *MAG* cis-acting element to drive transgene expression *in vivo*. The identification of the potential of the 0.3 kb *MAG* promoter fragment might be the most important result of the present study. The significance of this result is outstanding as this promoter fragment allows incorporation in the genome of self-complementary (sc) AAV vectors that inherently possess only half the packaging capacity yet have a superior transduction profile compared with single-stranded vectors (McCarty, 2008). Although not investigated here, the presence of a RNF10 site within all three MAG promoter fragments might enable transgene expression in Schwann cells. This would be an improvement over the *Mbp* promoter that lacks the Schwann cell-specific enhancers required for activity in the peripheral nervous system (Mathis et al., 2000).

In conclusion, the *MAG* promoter has superior features considering its human origin, small size and oligodendroglial selectivity in adults and neonates. Combined with its potential to sustain long-term transgene expression, the recombinant *MAG* promoter represents a valuable addition to the AAV toolbox.

## Author Contributions

Conceived and designed the experiments: MK and GvJ. Performed the experiments: GvJ, XW, RR, DF, CBK, AEH, ZHTS and MK. Analyzed the data: GvJ, DF and GDH. Wrote the article: MK and GvJ.

## Conflict of Interest Statement

The authors declare that the research was conducted in the absence of any commercial or financial relationships that could be construed as a potential conflict of interest.

## Footnotes

^1^https://genome.ucsc.edu

^2^http://pipeline.lbl.gov/cgi-bin/gateway2

^3^http://jaspar.genereg.net

^4^http://bioinfo.wilmer.jhu.edu/PDI/index.html

^5^http://www.gene-regulation.com/pub/databases.html
